# The Reinforced Ma–Griffith Method Combined with Minimally Invasive Small‐Incision Suture for Acute Achilles Tendon Rupture

**DOI:** 10.1111/os.13140

**Published:** 2021-12-22

**Authors:** Hao Yu, Fangyuan Wang, Jia Xie, Yunfeng Yao, Juehua Jing, Jun Li

**Affiliations:** ^1^ Department of Orthopaedics The Second Affiliated Hospital of Anhui Medical University Hefei China

**Keywords:** Achilles tendon, Minimally invasive surgery, Reinforced Ma–Griffith method, Rupture, Small incision

## Abstract

**Objective:**

To evaluate the treatment effects of the reinforced Ma‐Griffith method combined with a minimally invasive small incision(M‐G/MISI) in the treatment of acute Achilles tendon rupture.

**Methods:**

From January 2012 to January 2020, a retrospective study was carried out on thirty‐one patients with acute Achilles tendon ruptures that were treated using the M‐G/MISI. Patient with acute Achilles tendon rupture was operated on in the prone position. The M‐G/MISI begin with making a small incision to debride the stumps of ruptured tendon. Then M‐G/MISI was used to suture the distal and proximal Achilles tendons with the help of a epidural puncture needle and polydioxanone synthetic absorbable suture (PDS) Ⅱ line. Finally the stumps of ruptured tendon was reattached. After the surgery, the affected limb was fixed with either a plaster slab below the knee brace or a functional brace. Removal of plaster external fixation and partial weight‐bearing with crutches five weeks after the operation; Complete weight‐bearing nine weeks after the operation; jogging permitted 12 weeks after the operation; Patients were allowed to resume normal activities six months after the operation.

**Results:**

All 31 patients in this study were male. Nineteen of these patients had Achilles tendon rupture on the right lower extremity, while 12 had ruptures on the left lower extremity. The patients had a mean age of 33.35 ± 7.13 years (range, 18‐52 years). The mean operation time was 79.58 ± 22.67 minutes (range, 40‐167 minutes). The mean time from injury to operation was 4.19 ± 2.01 days (range, 1‐8 days), and the mean hospital stay was 9.87 ± 3.88 days (range, 5‐22 days). The new technique had a small incision with a mean length of 3.94 ± 1.82 cm (range, 2‐6 cm). The mean intraoperative blood loss was 16.77 ± 13.76 mL (range, 10‐50 mL) and the mean follow‐up time was 21.35 ± 10.18 months (range, 6‐50 months). No wound infection, fistula, skin necrosis, sural nerve damage, deep venous thrombosis or tendon re‐rupture was found. One year after the surgery, all patients reported 97.00 (range, 93‐100 points) AOFAS ankle‐hindfoot score points and the mean ATRS was 97.39 (range, 91‐100) points.

**Conclusion:**

The reinforced Ma‐Griffith method, combined with a minimally invasive small incision suture, is a simple, effective, minimally invasive technique and low‐cost surgical method for the treatment of acute Achilles tendon rupture.

## Graphical abstract

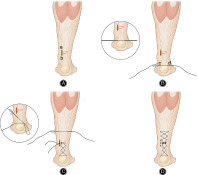

This study aimed to evaluate the treatment effects of the reinforced Ma–Griffith method combined with a minimally invasive small incision in the treatment of acute Achilles tendon rupture. I: Summary of patients' preoperative status. II: Reinforced Ma–Griffith method combined with a minimally invasive small‐incision technique. III: Summary of patients' postoperative recovery status (AOFAS and ATRS).

## Introduction

With the rapid development of society, human life is also changing. More and more people participate in sports activities, which increases the risk of injury. The Achilles tendon is the strongest tendon in the human body, but it is also one of the tendons most prone to injury and rupture. The Achilles tendon consists of the tendon parts of the gastrocnemius and soleus muscles which combine to form the strongest and largest tendon^1^ in the human body. Achilles tendon injuries are more common in men than in women^2^. Many studies^3^ have found that the incidence of acute Achilles tendon rupture has increased significantly. The most common site of injury is 2–6 cm above the calcaneal tubercle where the blood supply is very poor^4^. Contrarily, the latest study has found that most acute Achilles tendon ruptures occur 5–8 cm above the distal point of the calcaneus^5^. Angiography has confirmed Mayer's finding in 1916 that the blood supply to the tendon is from three areas: the musculotendinous junction, the osseotendinous junction, and the paratenon where the posterior tibial artery is situated. However, qualitative and quantitative histological analyses have shown that the Achilles tendon has a poor blood supply throughout its length. This has been determined by the small number of blood vessels per cross‐sectional area which do not vary significantly along the Achilles tendon length^6^.

At present, the treatment of acute Achilles tendon rupture includes surgical treatment and non‐operative treatment (that is, conservative treatment). Surgical treatment generally includes traditional enhanced or non‐enhanced repair, limited small‐incision surgery, percutaneous minimally invasive surgery, improved percutaneous repair, and the use of special minimally invasive surgical equipment (such as Achilles tendon stapler) to treat Achilles tendon rupture. There are many ways to treat acute Achilles tendon rupture; however, the best treatment remains controversial^7^. Conservative treatment is usually used in patients with severe underlying disease, in patients who do not wish to undergo surgery, and in patients with low indications for surgery. These patients have a greater postoperative risk than conservative treatment^8^. Surgical repair can shorten the fixed time. Furthermore, the effect of surgical treatment is good^9^. Compared with the nonoperative approach, open surgical repair of acute Achilles tendon rupture significantly reduces the risk of recurrence. However, the incidence of other complications has also increased parallel to surgery^10^. Minimally invasive and percutaneous repair methods are less invasive and can reduce the risk of soft tissue complications^11^. However, because the ruptured site of the Achilles tendon is not exposed, some percutaneous repair methods may lead to poor involution of the torn site and weak biomechanical resistance. This leads to an increased risk of re‐rupture and sural nerve injury^12^. In view of the existing problems related to traditional incision and minimally invasive repairs, we have proposed a new surgical method: the reinforced Ma–Griffith method combined with a minimally invasive small‐incision (M‐G/MISI) surgery. This method involves a percutaneous suture, limited incision, direct visualization, and a more reliable suture of the broken end of the Achilles tendon. At the same time, the method uses a lumbar puncture needle which greatly reduces the surgical cost for patients. In this study, we aimed to: (i) functionally and aesthetically repair acute Achilles tendon rupture; (ii) assess the long‐term clinical efficacy of M‐G/MISI with a follow‐up of at least 6 months; and (iii) discuss the surgical precautions, limitations, and further surgical development of acute rupture of Achilles tendon.

## Materials and Methods

We conducted a retrospective study on patients with Achilles tendon rupture admitted to the Second Affiliated Hospital of Anhui University between January 2012 to January 2020. A total of 31 patients were included in this study.

### Inclusion and Exclusion Criteria

The inclusion criteria were as follows: (i) diagnosis of acute closed Achilles tendon rupture after a comprehensive clinical physical examination and imaging; (ii) age ≥18 years; and, (iii) reinforced Ma–Griffith method combined with a minimally invasive small incision employed for treatment.

The exclusion criteria were as follows: (i) open Achilles tendon rupture; (ii) chronic pain in the Achilles tendon; (iii) calcaneal fracture; (iv) old Achilles tendon rupture; and (v) drugs have been injected locally into the Achilles tendon in the past.

### Surgical Equipment and Procedure

**Fig. 1:**
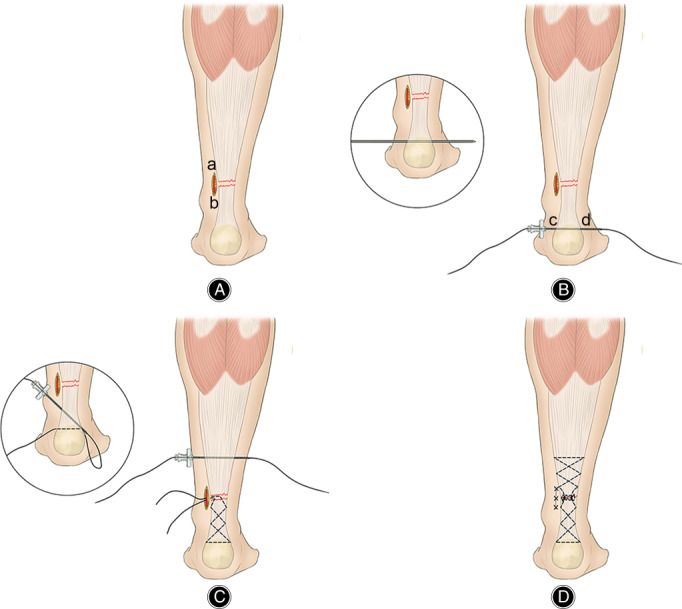
Schematic diagram: The black dotted line indicates the suture track of the inside of the tendon. (A) Make an incision of about 2 cm on the medial side of the Achilles tendon; (B) cd is the two symmetrical points of the distal end of the Achilles tendon. The Kirschner needle is used to pass through the cd with the help of an electric drill, followed by an epidural puncture needle along the cd through the Achilles tendon, and the PDS II thread passes through it; (C) The epidural puncture needle passes through the Achilles tendon along the planned route before operation, and then the PDS II thread passes through the epidural puncture needle; (D) As mentioned above, the ruptured Achilles tendon is sutured according to the planned route.

**Fig. 2:**
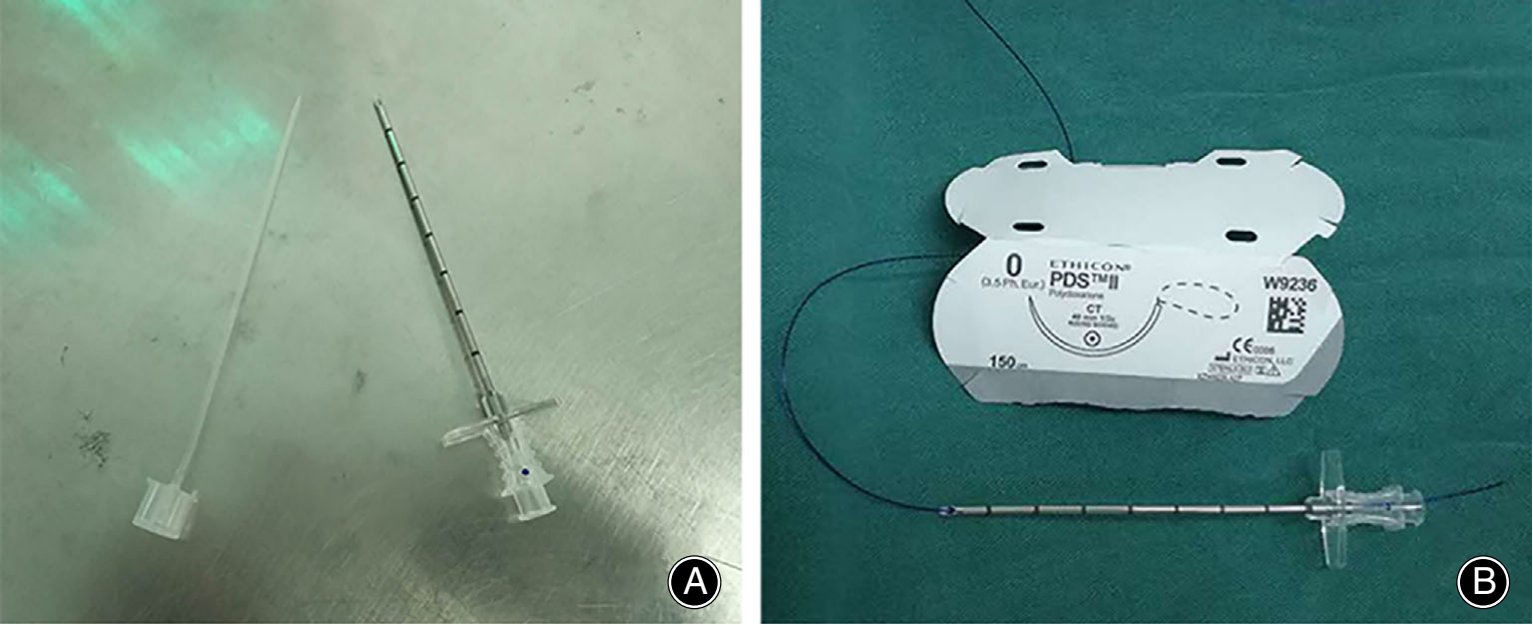
**(**A) Epidural puncture needle; (B) polydioxanone synthetic absorbable suture (PDS) II thread.

All operations were performed by an experienced orthopaedic surgeon. The M‐G/MISI technique (Fig. 1) was used. The patient was placed in a prone position and subjected to general anesthesia. First, touching the sunken end of the Achilles tendon, a 2–3 cm incision was made on the medial side of the Achilles tendon (Fig. 1A point a to point b). The skin was cut, the subcutaneous and peritendon tissues turned, and hematoma at the broken end removed. The broken end of the Achilles tendon was carefully exposed. Tissue forceps were used to organize the broken end and lengthen the incision if necessary. The M‐G/MISI was used to suture the distal and proximal Achilles tendons with the help of an epidural puncture needle and polydioxanone synthetic absorbable suture (PDS) II thread (Fig. 2). Drill holes along the calcaneus perpendicular to the Achilles tendon with a 2.0‐mm Kirschner needle at the distal end, the broken end was pulled with tissue forceps. The Kirschner needle was inserted at point c in Fig. 1B and drawn out at point d, so that the epidural puncture needle was crossed symmetrically through the broken end of the Achilles tendon until the epidural puncture needle finally pierced out at the broken end of the Achilles tendon. At the proximal end of the Achilles tendon, the broken end was pulled with tissue forceps, the tendon was crossed about 3 cm from the broken end, and the epidural puncture needle was inserted and pulled out so that the lines on both sides were equal. Throughout the process, the PDS II thread passes through the waist needle. Using the same method described above, the lumbar spinal needles were finally drawn out symmetrically at the medial and lateral side of the broken end of the Achilles tendon. With the ankle in a plantar flexion position, the distal and proximal PDS II thread were tightened. At the broken end of the Achilles tendon in the ab incision, the knot was fastened from the inside and outside of the Achilles tendon, and then an absorbable suture was used to strengthen the suture at the broken end.

### Postoperative Management

**Fig. 3:**
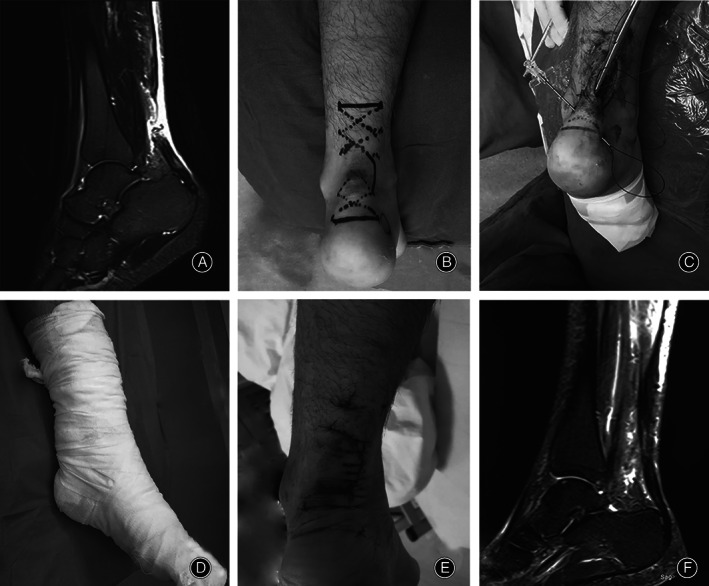
A 29‐year‐old male with a ruptured Achilles tendon because of playing basketball. (A) Preoperative left lower limb MRI (magnetic resonance imaging, Siemens 3.0T) showed rupture of Achilles tendon; (B) before the operation, plan the suture route; (C) during the operation, with the help of epidural puncture needle, the PDS II thread passes through the Achilles tendon; (D) after the operation, the plaster was fixed; (E) the picture of the surgical incision when changing the dressing after the operation; (F) postoperative MRI picture.

**Fig. 4:**
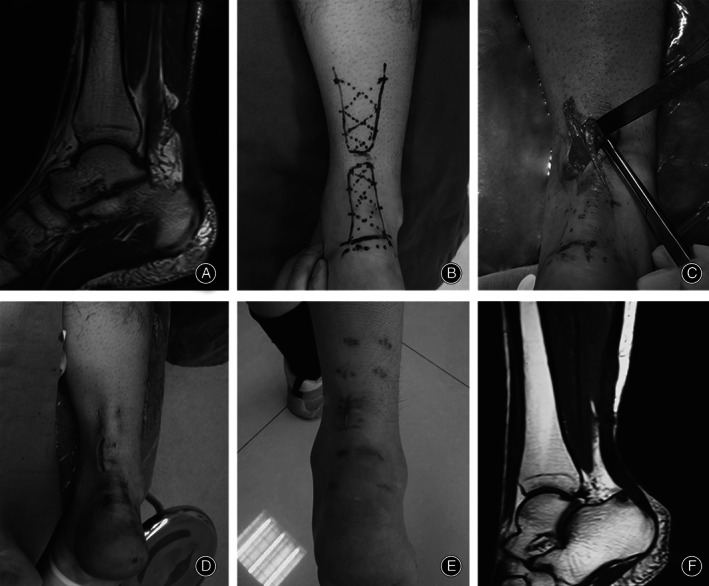
A 31‐year‐old male patient with ruptured Achilles tendon due to playing badminton. (A) Preoperative right lower limb MRI showed rupture of Achilles tendon; (B) before the operation, plan the suture route; (C) exposure of the broken end of the Achilles tendon during the operation; (D) before the incision is closed, a small incision can be seen; (E, F) postoperative MRI showed that the Achilles tendon had been repaired. During the follow‐up 6 months after operation, the incision was very small and the man returned to normal life.

**Fig. 5:**
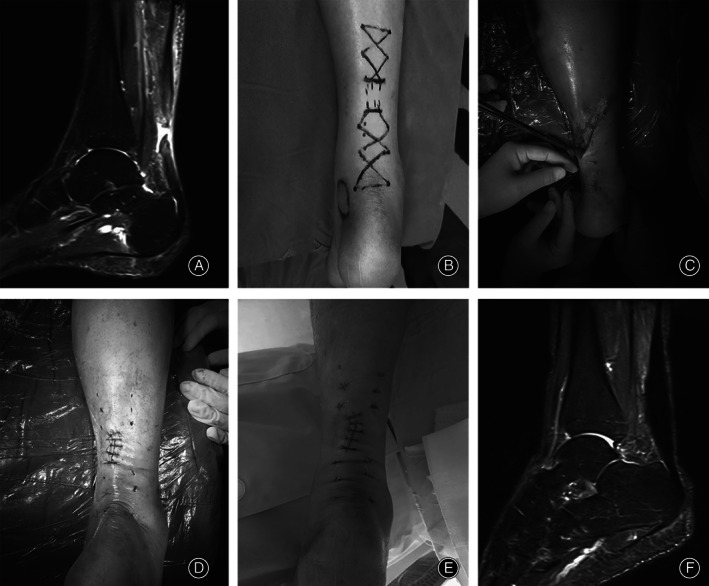
A 44‐year‐old middle‐aged man suffered a rupture of the Achilles tendon after a sprain. (A) Preoperative right lower limb MRI showed rupture of Achilles tendon; (B) draw the operation route before operation; (C) Suture Achilles tendon with the help of epidural puncture needle; (D) Close the incision and complete the operation; (E) On the second day after operation, the surgical incision was small and the patient was very satisfied; (F) Postoperative MRI showed that the Achilles tendon anastomosed well, and during the postoperative follow‐up, it was found that the patient returned to normal work and sports.

After the operation, the affected limb was raised. Symptomatic treatments such as detumescence and analgesia were provided to patients. The knees were stabilized using a tubular plaster below the affected knee or a functional brace. Patients were allowed to walk on crutches as long as the affected limb did not bear weight. The hip and knee joints were strengthened using functional exercises such as normal flexion and extension of toes. These exercises actively prevented venous thrombosis of the lower extremities. Hip and knee joints were exercised within 2 weeks after the operation. Stitches were removed during postoperative week 2. Flexion and extension of toes were performed within 5 weeks after the operation. In postoperative week 5, external plaster fixation was removed and partial weight‐bearing of crutches was undertaken. In postoperative week 9, complete weight‐bearing was achieved. Jogging could be done 12 weeks after the operation. Finally, at 6 months after the operation, the patient resumed normal activities (Figs 3, 4, 5).

### Follow‐Up and Data Collection

During the patients' postoperative reexamination, the chief surgeon tested for recovery, patients' scores were recorded accordingly. The evaluation consisted of the patient's sex, affected side, age, operation time, incision length, blood loss during operation, hospital stay, injury to operation time, American Orthopaedic Foot and Ankle Society (AOFAS) score^13^, ^14^, and the Achilles tendon rupture score (ATRS)^15^, ^16^.

#### American Orthopaedic Foot and Ankle Society Ankle‐Hindfoot Score

The AOFAS developed four rating systems to provide a standard method of reporting the clinical status of the ankle and foot. The systems incorporated both subjective and objective factors into numerical scales to describe function, alignment, and pain. The maximum score is 100 points (best possible outcome). Total scores of <50, 50–74, 75–89, and 90–100 indicate poor, fair, good, and excellent outcomes, respectively.

#### Achilles Tendon Rupture Score

The ATRS is a patient‐reported instrument with high reliability, validity, and sensitivity for measuring outcomes after treatment in patients with a total Achilles tendon rupture. ATRS was used to evaluate the limitation of calf, Achilles tendon, and foot movement after Achilles tendon injury, and systematically evaluated 10 problems such as pain, daily activity, medium‐intensity exercise, and high‐intensity exercise after Achilles tendon injury. The full score of each item was 10, and the degree of functional limitation of Achilles tendon was classified according to slight, moderate, serious, and severe.

## Results

### Sample Characteristics of Patients

All 31 patients in this study were male. Nineteen of these patients had Achilles tendon rupture on the right lower extremity, while 12 had ruptures on the left lower extremity (Table 1). The patients had a mean age of 33.35 ± 7.13 years (range: 18–52 years) (Table 2). The operation time was 79.58 ± 22.67 minutes (range: 40–167 minutes). The time from injury to operation was 4.19 ± 2.01 days (range: 1–8 days), and the hospital stay was 9.87 ± 3.88 days (range: 5–22 days). The new technique had a small incision with a mean length of 3.94 ± 1.82 cm (range: 2–6 cm). The intraoperative blood loss was 16.77 ± 13.76 mL (range, 10–50 mL) and the follow‐up time was 21.35 ± 10.18 months (range: 6–50 months).

**TABLE 1:** Sample characteristics of patients

Age	Gender		Injury side		Time from jury to operation (days)	Operation time
	Male	Female	Left	Right		
33.35 ± 7.13	31	0	12	19	4.19 ± 2.01	79.58 ± 22.67

**TABLE 2:** Sample characteristics of patients

Length of incision (cm)	Blood loss of surgery (mL)	Hospitalization time (days)	Follow‐up time (months)
3.94 ± 1.82	16.77 ± 13.76	9.87 ± 3.88	21.35 ± 10.18

### AOFAS

At the 3rd month of follow‐up, the patient's AOFAS score increased from 47.42±5.14 points (range, 40‐55 points) to 91.23±2.03 points (range, 85‐95 points). During the 6th month of follow‐up, the AOFAS score was 97.00 ± 2.67 points (range, 93‐100 points).

### ATRS

Similarly, during the 3rd month of follow‐up, the ATRS preoperative score increased from 47.26 ± 4.97 points (range: 40‐55 points) to 91 ± 1.91 points (range: 88‐95 points). During the 6th month of follow‐up, the ATRS score was 97.39 ± 2.85 points (range: 91‐100 points) (Table 3).
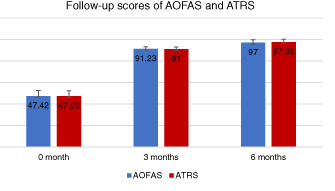


**TABLE 3:** Sample follow‐up characteristics of patients

AOFAS0	AOFAS3	AOFAS6	ATRS0	ATRS3	ATRS6
47.42 ± 5.13*	91.23 ± 2.03	97.00 ± 2.67	47.26 ± 4.97	91.00 ± 1.91	97.39 ± 2.85

* Values are expressed as the Mean ± (SD).

### Complication

All patients have a successful acute Achilles tendon rupture repair using the reinforced Ma–Griffith method combined with a minimally invasive small‐incision suture. There were no operation‐related complications such as deep wound infections or sural nerve injuries.

## Discussion

### Different Surgical Methods for Acute Achilles Tendon Rupture

In the past, compared with minimally invasive surgery, traditional incision repair is more stable. However, infections are more likely to occur with traditional repair. In a study of 815 patients^17^, the rate of sural nerve injury in the percutaneous group was significantly higher than that in the control group, and there was no significant difference in the rate of rerupture. In the percutaneous group, the operation time was shorter and the AOFAS was higher.

To reduce incisional complications, Ma and Griffith proposed the Ma‐Griffith method in 1977^18^ method. This method reduced the risk of infection, avoided long incisions, and avoid delayed healing, However, repeated insertions of the Achilles tendon knots could easily lead to nerve injury and knot compression and the subsequent percutaneous suture strength has been questioned. More recent studies have shown that re‐rupture rate and the rate of sural nerve injury were as high as 8% and 13%, respectively^19^. Cretnik *et al*.^20^ compared 132 patients in the percutaneous repair group and 105 patients in the open repair group. The main complications in the percutaneous repair group were significantly less than those in the open repair group (4.5% *vs* 12.4%), especially for necrosis (0% *vs* 5.6%). The total number of complications was also considerably less (9.7% *vs* 21%). Percutaneous minimally invasive surgery has the advantages of a small incision and a shorter operation time. However, sural nerve injury is a complication that cannot be ignored. Compared with open surgery, percutaneous minimally invasive surgery has significantly reduced the incidence of incision complications. It has also yielded higher patient satisfaction.

The technique of minimally invasive Achilles tendon repair was first proposed by Kakiuchi^21^ and it combined the advantages of the open and percutaneous techniques. A needle is inserted into both ends of the Achilles tendon through a small incision (approximately 2–4 cm) and sutured percutaneously. This method was later improved by Rippstein^22^ and Assal^23^. Valente^24^ treated 35 patients using an Achilles tendon device. There were no complications such as postoperative infection or recurrence. Patients returned to normal activity within 2 months. The small‐incision minimally invasive repair technique represented by Achilles tendon technology not only achieved relatively accurate alignment of the Achilles tendon, but also reduced surgical trauma and postoperative adhesions. However, the equipment needed for this operation placed a financial burden on patients.

### Functionally and Aesthetically Repair Acute Achilles Tendon Rupture

Combining the advantages and disadvantages of the aforementioned strategies, we developed a novel method in the present study: the reinforced Ma–Griffith method combined with minimally invasive small‐incision suture. Based on the limited incisions during percutaneous suturing, an epidural puncture needle was used rather than the expensive equipment for minimally invasive Achilles tendon surgery. This new surgical method not only reduced complications such as incision infections, but also made the broken end of the Achilles tendon suture firm. Because an epidural puncture needle was used, the cost of the operation has been greatly reduced. At the same time, the length of the incision is very small and all patients were satisfied.

### Long‐Term Clinical Efficacy of M‐G/MISI

During the follow‐up period, no wound infection, fistula, skin necrosis, sural nerve damage, deep venous thrombosis, or tendon re‐rupture was found. One year after the surgery, all patients reported 97.00 (range: 93–100 points) AOFAS ankle‐hindfoot score points and the mean ATRS was 97.39 (range, 91–100) points. All data suggest that M‐G/MISI is a simple, minimally invasive and effective surgical approach for the treatment of acute Achilles tendon rupture.

### Surgical Experiences

(i) The chief surgeon should master the course of the sural nerve and avoid injury as far as possible; (ii) Carefully mark the rupture site of the Achilles tendon before operation, and it can be located by ultrasound, if possible; (iii) The Kirschner needle drilling on the calcaneus should be located correctly, not too high or too low, and you can try to pull after threading; (iv) Be sure to protect the soft tissue around the cut, especially the tissue around the tendon, and cover the knot at the broken end of the Achilles tendon with peri‐tendon tissue when closing the incision; (v) At the broken end of the Achilles tendon, when the knot is tied tightly from the inside and outside, the plantar ankle flexion must be closed, and after a strong suture, Thompson test is performed to check whether the continuity of the Achilles tendon is restored; (vi) External fixation should be achieved with plaster after operation, and rehabilitation exercise conducted under the guidance of doctors.

### Limitations

This study has some limitations. First, the patient population was limited to a certain area instead of being multicenter. Second, all the patients who participated were male. Third, the study had a small number of patients. Thus, it may not have been representative of the general population. This study is not only simple and effective, but it was also conducted at a low cost. Further expanding the scope of the study will help to verify the efficacy of this surgical method and popularize it in the future.

### Conclusion

In conclusion, the reinforced Ma‐Griffith method combined with minimally invasive small incision suture(M‐G/MISI) can well suture the acute ruptured Achilles tendon, and the operation is simple and inexpensive.

## Author Contribution

Jun Li designed the study. Juehua Jing and Yunfeng Yao participated in the design of the study. Hao Yu, Fangyuan Wang, and Jia Xie enrolled patients. Hao Yu performed literature search. Hao Yu wrote the initial draft. Hao Yu conducted statistical analysis. Jun Li, Juehua Jing, and Yunfeng Yao provided comments and prepared the final version of the manuscript for publication. All authors read and approved the final manuscript.
